# Predictive value of multivariate models combining CT-based extracellular volume fraction with clinicopathological parameters for preoperative detection of occult lymph node metastasis in gastric cancer

**DOI:** 10.1186/s13244-025-02172-6

**Published:** 2025-12-22

**Authors:** Shuangshuang Sun, Lin Li, Mengying Xu, Song Liu, Zhengyang Zhou

**Affiliations:** 1 Department of Radiology, Nanjing Drum Tower Hospital, Affiliated Hospital of Medical School, Nanjing University, Nanjing, China; 2 Department of Pathology, Nanjing Drum Tower Hospital, Affiliated Hospital of Medical School, Nanjing University, Nanjing, China

**Keywords:** Stomach neoplasms, Lymph node metastasis, Extracellular volume, Tomography (x-ray computed)

## Abstract

**Objectives:**

To develop multivariate models for preoperative detection of occult lymph node (LN) metastasis in gastric cancer (GC), by integrating CT-based extracellular volume (ECV) fraction and clinicopathological features, and further evaluate the prognostic value of the combined model.

**Materials and methods:**

This retrospective study included 129 GCs with the N (−) group (*n* = 49) and the N (+) group (*n* = 80). The preoperative CT parameters (including ECV fraction), WHO types and differentiation degree based on endoscopic pathological, and 4 hematological indices were assessed. The diagnostic performance of multivariate models was evaluated by receiver operating characteristic curve analysis.

**Results:**

The N (+) group demonstrated significantly higher proportions of poorly cohesive carcinoma and poor differentiation based on endoscope (both *p* < 0.001). Significantly higher CT-measured tumor area and ECV fraction were seen in the N (+) group (*p* < 0.001 and *p* = 0.008, respectively), and a significantly higher proportion of ECV value > 50% in the N (+) group (*p* = 0.001). The clinicopathological model, CT parameters model, and combined model yielded areas under the curves of 0.768, 0.774, and 0.843, respectively. The combined model with the high-risk group revealed a significantly shorter median recurrence-free survival compared to the low-risk group (*p* = 0.008).

**Conclusion:**

The proposed preoperative combined model exhibited a promising performance for early predicting occult LN metastasis and stratifying postoperative recurrence risk in GC, by integrating CT-based ECV fraction and clinicopathological features.

**Critical relevance statement:**

The CT-based ECV preoperative model could potentially provide valuable clinical reference for making clinical strategies in GC.

**Key Points:**

- It is a great challenge for clinicians to evaluate occult lymph node (LN) status in gastric cancer (GC).
- The N (+) group demonstrated higher CT-based extracellular volume (ECV) fractions and tumor area, and higher proportions of poorly cohesive carcinoma and poor differentiation.
- This model helped preoperative detection of occult LN metastasis and stratifying postoperative recurrence risk in GC.

**Graphical Abstract:**

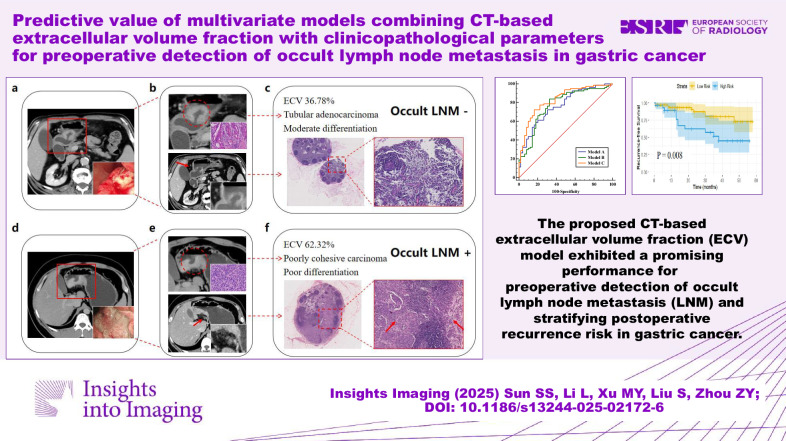

## Introduction

Gastric cancer (GC) is the fifth most prevalent cancer worldwide, accounting for the fifth leading cause of cancer-related deaths [1]. Lymph node (LN) metastasis, one of the most predominant metastatic forms in GC, has a profound impact on clinical therapeutic strategies and prognosis evaluation [2–4]. In clinical practice, preoperative LN assessment in GCs mainly relies on CT, which predominantly utilizes LN size to evaluate LN status. However, even if the LNs are not enlarged on preoperative CT, some LNs are still confirmed metastatic [2, 5]. Therefore, it is a great challenge for clinicians to evaluate occult LN in GC.

The extracellular volume (ECV) fraction, encompassing both intravascular and extravascular spaces [6–8], has emerged as a quantitative biomarker for evaluating extracellular matrix (ECM) volume, which reflects the tumor microenvironment [7, 9]. In recent years, ECV has been increasingly explored in the diagnosis and evaluation of tumors [6, 7, 9–11]. For instance, Li et al [7] utilized CT-ECV to predict the preoperative pathologic grade of rectal cancer. Chen et al [6] confirmed the effectiveness of CT-ECV in predicting tumor regression grade after preoperative immunochemotherapy in advanced GC.

Preoperative endoscopic pathology of GC patients can be routinely obtained, which provides tumor characteristics on the cell level. Notably, different histopathological subtypes of GCs exhibit differential LN metastasis risks [12–14]. Poorly cohesive carcinoma, a distinct subtype of GCs, is characterized by isolated or small aggregates of discohesive carcinoma cells with high risks of infiltration [15], which contains signet ring cell carcinoma and others. Kao et al [14] analyzed the clinicopathological characteristics of 2971 GC patients, and demonstrated that signet ring cell carcinoma had more LN metastasis compared to non-signet ring cell carcinoma in T2-T4 GCs.

Most previous studies mainly focused on employing either CT parameters or clinicopathological features alone in predicting LN metastasis of GC, instead of integrating them. Thus, we aimed to develop a preoperative combined model integrating CT parameters (including ECV fraction) and endoscopic pathological features to predict occult LN metastasis in GC, and further evaluate the prognostic value of the combined model.

## Materials and methods

### Patients

This retrospective study gained approval from the institutional review board and a waiver of informed consent (No. 2024-081-02).

**Fig. 1:**
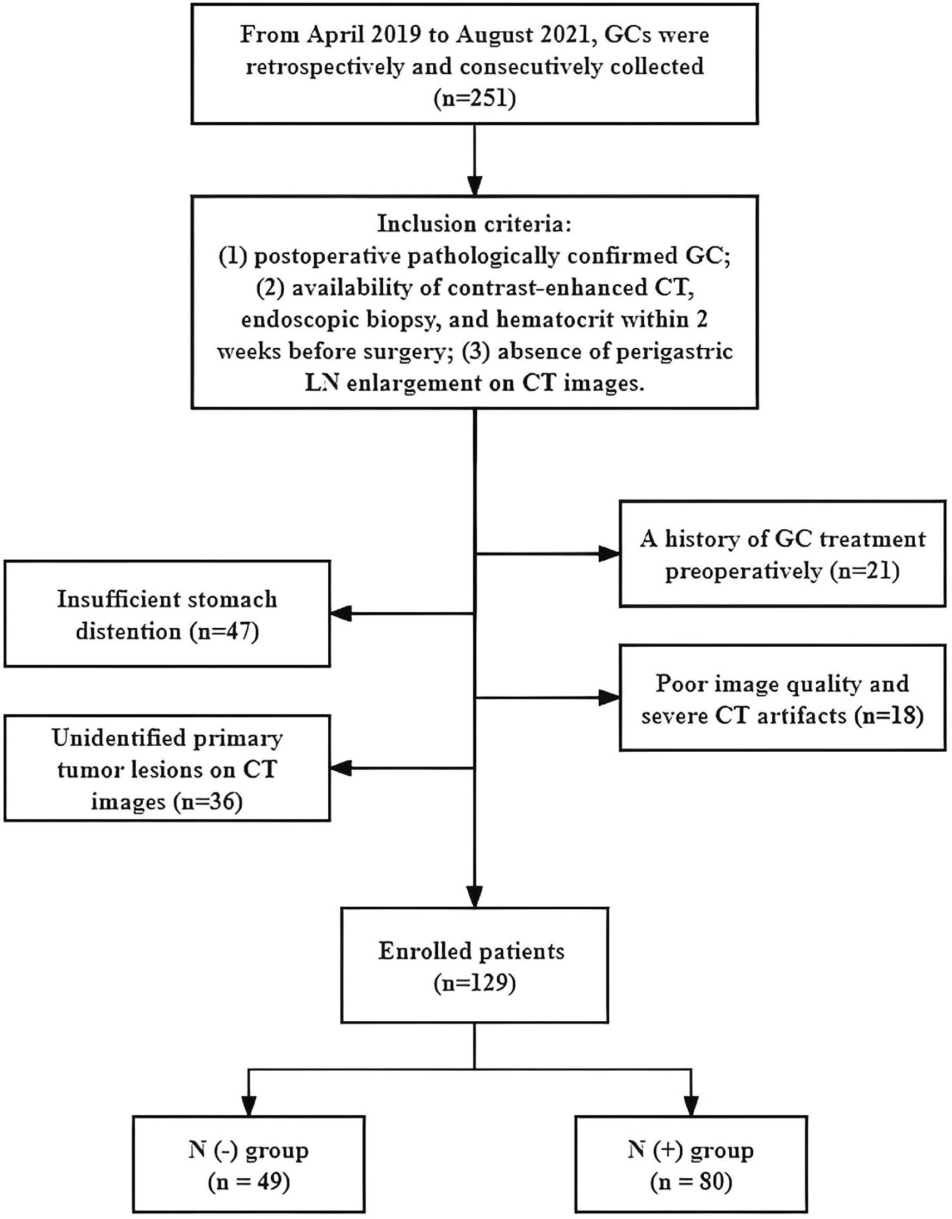
The flowchart of the enrolled patients in this study. GC, gastric cancer; LN, lymph node

**Fig. 2:**
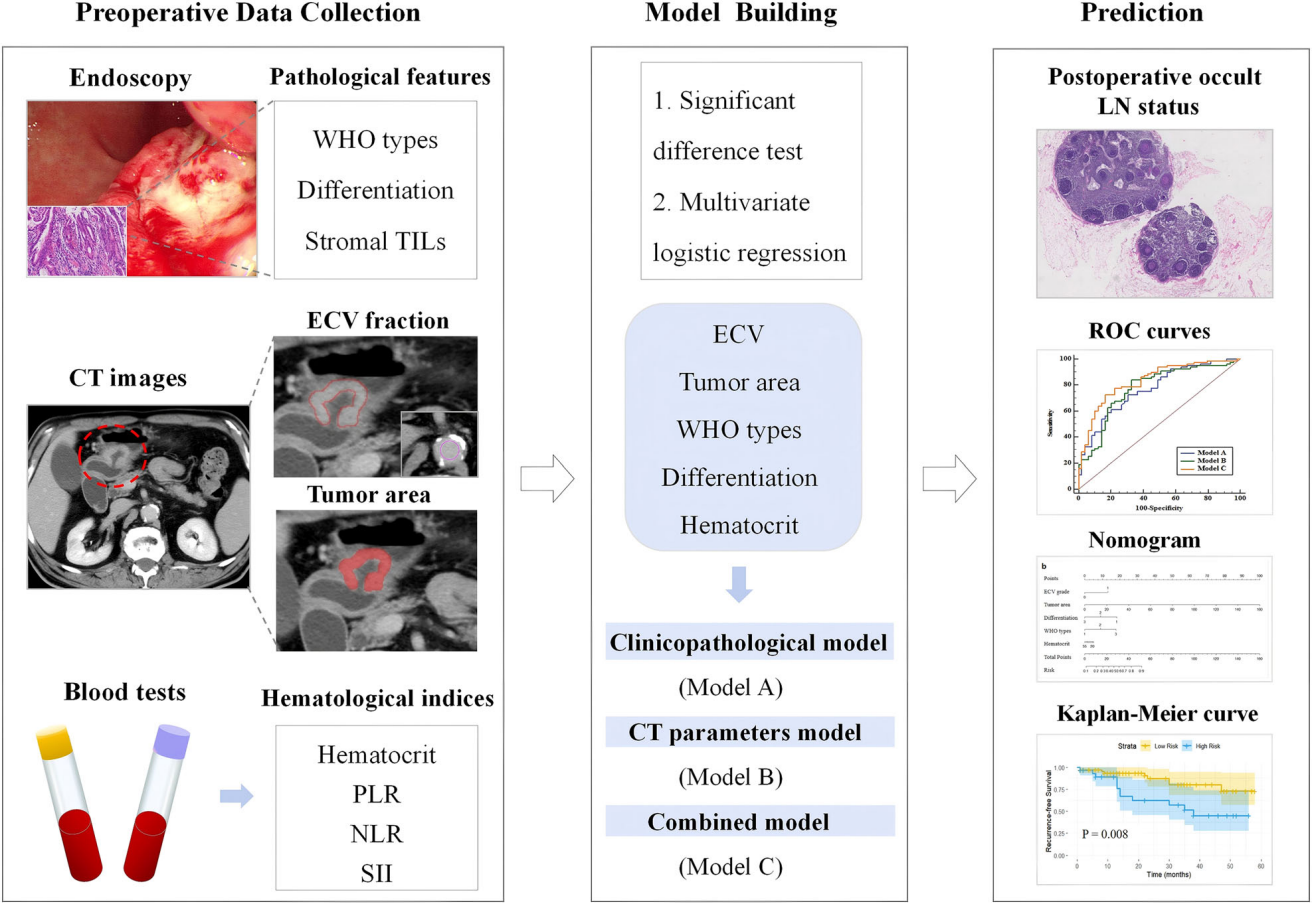
The workflow of this study. PLR, platelet-lymphocyte ratio; NLR, neutrophil-lymphocyte ratio; SII, systemic immune-inflammation index; TILs, tumor-infiltrating lymphocytes; ECV, extracellular volume; LN, lymph node; ROC, receiver operating characteristic curve

From April 2019 to August 2021, GCs in our hospital were retrospectively and consecutively collected. The inclusion criteria were as follows: (1) postoperative pathologically confirmed GC; (2) availability of contrast-enhanced CT, endoscopic biopsy, and hematocrit within 2 weeks before surgery; (3) absence of perigastric LN enlargement on CT images (LN with short diameter ≥ 10 mm on axis CT was defined as LN enlargement) [5]. The exclusion criteria were as follows: (1) a history of GC treatment preoperatively; (2) insufficient stomach distention; (3) poor image quality and severe CT artifacts; (4) unidentified primary tumor lesions on CT images. The flowchart of the enrolled patients is presented in Fig. 1. The overall workflow of this study is displayed in Fig. 2.

### Hematological indices

Hematological indices, including hematocrit, absolute lymphocyte, neutrophil, and platelet counts, were collected. Then, the neutrophil-lymphocyte ratio (NLR), Platelet-lymphocyte ratio (PLR), and systemic immune-inflammation index (SII) were calculated [4, 16, 17] (Supplementary Material).

### Endoscopic pathological evaluation

WHO types and histological differentiation degree based on preoperative endoscopic biopsy were retrospectively evaluated by a pathologist (with 10 years of experience in the pathological diagnosis of the digestive system), referring to the 2019 WHO Classification of Tumors of the Digestive System [15]. WHO types include tubular adenocarcinoma, poorly cohesive carcinoma, and other types. Differentiation degree was classified as poor, well, and moderate. Tubular adenocarcinoma consists of enlarged or dilated or slit-like branching tubules, while poorly cohesive carcinoma is composed of isolated or small arranged tumor cells without forming well-formed glands [15, 18]. Furthermore, the stromal tumor-infiltrating lymphocytes (TILs) percentage was assessed [19] and divided into ≤ 10% (lower level), 11–40% (moderate level), and > 40% (higher level).

### CT technique

CT scans were performed with the 64-row scanner (uCT 780, United Imaging). The arterial phase, venous phase, and delayed phase (DP) were obtained at 40 s, 70 s, and 180 s after contrast agent injection, respectively. The details of CT acquisition parameters were displayed in the Supplementary Material.

### Image analysis

CT images were assessed independently by a radiologist (with 6 years of abdominal diagnosis experience), blinded to clinicopathological information except for the general tumor location. Another Radiologist (with 12 years of abdominal diagnosis experience) repeated the above procedure to assess the interobserver reproducibility.

Regions of interest (ROIs) were drawn at the largest slice of the GC lesion and manually segmented along the edge of the lesion for CT values measured, avoiding adjacent vessels, gastric cavity, and necrosis [11]. The mean CT attenuation values of the tumor were recorded in the non-enhanced and DP. Tumor ROIs were delineated on 5-mm slice CT images, with reference to arterial phase, venous phase, and multi-planar reconstruction images. At the same time, the tumor area of each patient was recorded in DP.

### ECV fraction analysis

ECV fraction (%) was calculated utilizing the following formula:

ECV fraction (%) = (1 − hematocrit) × (ΔHU_tumor_/ΔHU_artery_) × 100% [6, 9, 20, 21]

where ΔHU_tumor_ and ΔHU_artery_ represent the CT attenuation values of the tumor and the blood pool (abdominal aorta) in DP minus the corresponding CT values in unenhanced, respectively.

The CT values of the abdominal aorta were measured by circular ROIs in the unenhanced and DP. Aortic ROIs were drawn at the same level of tumor ROIs as possible, so as to standardize measurements and minimize variability related to cardiac function and hemodynamics [10]. Circular ROIs were delineated as large as possible, avoiding any calcification and thrombus-attached vessel wall [6].

### Pathological assessment after surgery

In our study, all patients underwent standard gastrectomy with D2 lymphadenectomy. The total number of retrieved LNs ranged from 11 to 63, with a median of 24 LNs per patient.

After surgery, all gastric specimens were processed according to standard pathological procedures. Referring to the 8th American Joint Committee on Cancer classification [22], the pathological LN status was retrospectively recorded. GCs were divided into two groups: N (−) and N (+).

### Follow-up

Follow-up data after surgery of each patient were collected retrospectively, mainly based on imaging examinations. Recurrence-free survival (RFS) was defined as the time interval from surgery to the first date of recurrence, metastasis, or last follow-up [23, 24]. The end of the follow-up date was December 31, 2024.

### Statistical analysis

Continuous and categorical variables were analyzed using Mann–Whitney U test, chi-squared test, or Fisher’s exact test as appropriate. Starting with the statistically significant (*p* < 0.05) variables in univariate analysis, multivariate binomial logistic regression analysis based on an enter method was used to build the multivariate model for predicting occult LN metastasis. The Hosmer-Lemeshow test was applied to measure the goodness of fit. Diagnostic performance of prediction models was assessed by the area under the curve (AUC), and pairwise comparisons of AUCs between different models were performed by DeLong test. A nomogram was constructed based on the multivariate logistic regression analysis.

Interobserver agreement for quantitative measurements was evaluated by the intraclass correlation coefficient (ICC) and Bland–Altman analysis. RFS were estimated by Kaplan–Meier method and compared by log-rank test. The optimal cut-point for the prognostic model was obtained with X-tile software (version 3.6.1; Yale University School of Medicine, New Haven, Conn) [25, 26]. Model performance was assessed utilizing Harrell’s concordance index (C-index). To assess and adjust for potential optimism, we conducted bootstrap internal validation with 1000 repetitions.

All statistical analyses were conducted by SPSS (version 22.0), MedCalc Statistical Software (version 11.4.2.0), or R software (version 3.5.2). A two-tailed *p* < 0.05 indicated statistical significance.

## Results

This study finally enrolled 129 GC patients (male, 84; female, 45; median age, 64 years; age range, 30–91 years), divided into N (−) group (*n* = 49) and N (+) group (*n* = 80).

### Baseline demographic and postoperative pathological characteristics

Table 1 summarizes the baseline demographic and postoperative pathological characteristics of the N (−) and N (+) groups. No significant differences were found in gender and age between the two groups (both *p* > 0.05). The proportion of T3-4, poor differentiation, lymphovascular invasion (LVI) positive, and neural invasion (PNI) positive was significantly higher in the N (+) group compared to the N (−) group (all *p* < 0.001). A significantly higher proportion of diffuse type was seen in the N (+) group (*p* < 0.001).

**Table 1:** Demographic data and postoperative pathological characteristics of N (−) and N (+) groups

Characteristics	Total patients (*n* = 129)	N (−) group (*n* = 49)	N (+) group (*n* = 80)	*p*-value
Demographic data				
Gender				0.426
Male	84 (65.12%)	34 (69.39%)	50 (62.50%)	
Female	45 (34.88%)	15 (30.61%)	30 (37.50%)	
Age (years)				0.177
≤ 60	49 (37.98%)	15 (30.61%)	34 (42.50%)	
> 60	80 (62.02%)	34 (69.39%)	46 (57.50%)	
Postoperative pathology				
Major location				0.212
Cardia	48 (37.21%)	23 (46.94%)	25 (31.25%)	
Body	37 (28.68%)	12 (24.49%)	25 (31.25%)	
Antrum	44 (34.11%)	14 (28.57%)	30 (37.50%)	
T stage				< 0.001*
1–2	49 (37.98%)	35 (71.43%)	14 (17.50%)	
3–4	80 (62.02%)	14 (28.57%)	66 (82.50%)	
Lauren classification^a^				< 0.001*
Intestinal type	59 (46.46%)	35 (71.43%)	24 (30.77%)	
Diffuse type	32 (25.20%)	1 (2.04%)	31 (39.74%)	
Mixed type	36 (28.34%)	13 (26.53%)	23 (29.49%)	
Lymphovascular invasion				< 0.001*
Absent	66 (51.16%)	38 (77.55%)	28 (35.00%)	
Present	63 (48.84%)	11 (22.45%)	52 (65.00%)	
Neural invasion				< 0.001*
Absent	51 (39.53%)	31 (63.265%)	20 (25.00%)	
Present	78 (60.47%)	18 (36.735%)	60 (75.00%)	
Differentiation				< 0.001*
Poor	90 (69.77%)	22 (44.90%)	68 (85.00%)	
Moderate or well	39 (30.23%)	27 (55.10%)	12 (15.00%)	

Data in parentheses are percentages

* *p* < 0.05 with chi-squared test or Fisher’s exact test (*n* < 5)

^a^ Not applicable for two patients

### Hematological indices, endoscopic biopsy, and CT parameters

#### Hematological indices

The value of hematocrit in the N (+) group was significantly lower than that in the N (−) group (*p* = 0.041, Table 2). However, no significant differences were found in the values of PLR, NLR, and SII between the two groups (all *p* > 0.05).

**Table 2:** Univariate analysis of hematological indices, endoscopic biopsy, and CT parameters between N (−) and N (+) groups

Parameters	N (−) group (*n* = 49)	N (+) group (*n* = 80)	*p*-value
Hematological indices			
Hematocrit (%)	38.60 (35.20–41.80)	36.20 (31.60–40.00)	0.041*
PLR	136.11 (80.00–166.15)	141.43 (99.71–188.15)	0.168
NLR	2.08 (1.67–2.80)	1.93 (1.43–2.70)	0.645
SII	392.00 (292.56–536.82)	420.50 (284.46–631.89)	0.578
Endoscopic biopsy			
WHO types			< 0.001*
Tubular adenocarcinoma	43 (87.76%)	44 (55.00%)	
Poorly cohesive carcinoma	4 (8.16%)	25 (31.25%)	
Other types	2 (4.08%)	11 (13.75%)	
Differentiation degree			< 0.001*
Poor	11 (22.44%)	47 (58.75%)	
Moderate	19 (38.78%)	26 (32.50%)	
Well	19 (38.78%)	7 (8.75%)	
Stromal TILs percentage			0.420
≤ 10%	23 (46.94%)	44 (55.00%)	
11–40%	15 (30.61%)	25 (31.25%)	
> 40%	11 (22.45%)	11 (13.75%)	
CT parameters			
Tumor area (cm^2^)	8.63 (5.20–21.75)	25.50 (16.62–39.05)	< 0.001*
ECV (%)	38.19 (32.32–44.84)	44.97 (34.82–56.17)	0.008*
ECV grade			0.001*
≤ 50%	42 (85.71%)	46 (57.50%)	
> 50%	7 (14.29%)	34 (42.50%)	

Continuous variables are presented as the median (1st quartile, 3rd quartile), and categorical variables in parentheses are percentages

*PLR* platelet-lymphocyte ratio, *NLR* neutrophil-lymphocyte ratio, *SII* systemic immune-inflammation index, *TILs* tumor-infiltrating lymphocytes, *ECV* extracellular volume

* *p* < 0.05 with Mann–Whitney U test in continuous variables and with chi-square test or Fisher’s exact test (*n*＜5) in categorical variables

#### Endoscopic biopsy

As shown in Table 2, the proportion of poorly cohesive carcinoma was significantly higher in the N (+) group compared to that in the N (−) group (*p* < 0.001). A significantly higher proportion of poor differentiation was seen in the N (+) group (*p* < 0.001). There was no significant difference in stromal TILs percentage between the two groups (*p* > 0.05).

#### Tumor area and ECV

**Fig. 3:**
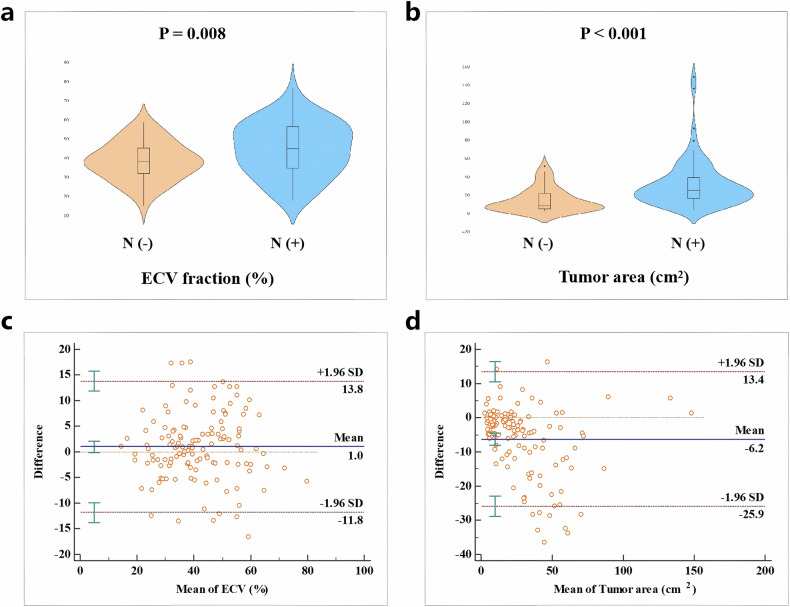
**a**, **b** The violin plots show the distribution of extracellular volume (ECV) fraction and tumor area in the N (−) and N (+) groups. The ECV fraction and tumor area in the N (+) group were significantly higher than those in N (−) group. **c**, **d** Bland–Altman plots of ECV fraction and tumor area show good interobserver agreement between two radiologists. The majority of data points fell within the 1.96 standard deviation, and the mean biases of ECV and tumor area between radiologists 1 and 2 were 1.0 and −6.2, respectively

**Fig. 4:**
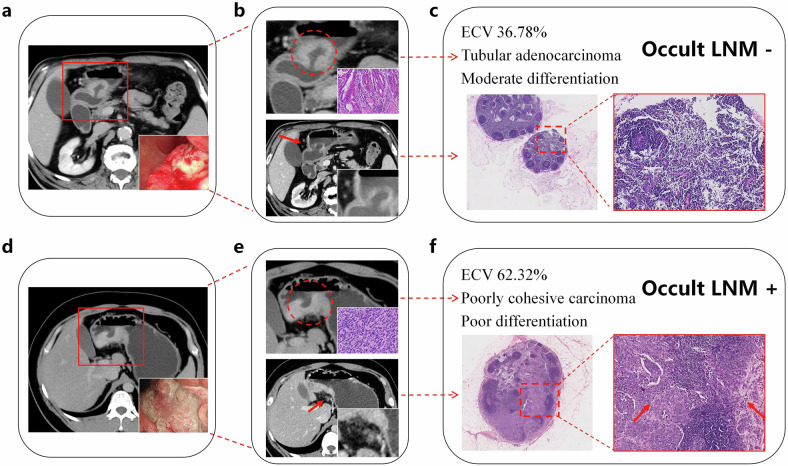
CT images and photomicrographs of occult lymph node (LN) metastasis negative and positive gastric cancer (GC). **a**–**c** A 73-year-old man with occult LN metastasis negative GC. **a** CT indicates a lesion in the antrum of the stomach. The lower right corner of the CT image was the endoscopic image of this lesion. **b** The upper images showed an enlarged view of the lesion and a specimen with Hematoxylin and eosin (H&E) staining based on endoscopic biopsy (the lower right corner), indicating a tubular adenocarcinoma with moderate differentiation. The lower CT image indicated non-enlarged LNs surrounding the lesion. **c** The extracellular volume (ECV) fraction was 36.78% in this patient, and H&E staining of a postoperative specimen (original magnification × 20 and original magnification × 200) confirmed LN metastasis (−). **d**–**f** A 38-year-old man with occult LN metastasis positive GC. **d** CT indicates a lesion mainly located in the antrum. The lower right corner of the CT image was the endoscopic image of this lesion. **e** The upper images showed an enlarged view of the lesion and a specimen with H&E staining based on endoscopic biopsy (the lower right corner), indicating a poorly cohesive carcinoma with poor differentiation. The lower CT image indicated non-enlarged LNs surrounding the lesion. **f** The ECV fraction was 62.32% in this patient, and H&E staining of a postoperative specimen (original magnification × 20 and original magnification × 200) confirmed LN metastasis (+). The arrow shows the areas of tumor infiltrating the LN

The values of tumor area and ECV fraction based on CT in the N (+) group were significantly higher than those in the N (−) group (*p* < 0.001 and *p* = 0.008, respectively) (Table 2) (Fig. 3a, b). We further divided the ECV values into two grades (≤ 50% and > 50%), indicating a significantly higher proportion of ECV values > 50% in the N (+) group (*p* = 0.001). The representative CT images and photomicrographs of occult LN metastasis negative and positive GC are depicted in Fig. 4.

### Development and performance of multivariate models

Multivariable logistic regression revealed ECV grade was an independent predictor of occult LN metastasis (OR = 3.119, 95% CI: 1.084–8.978, *p* = 0.035). Tumor area, WHO types and differentiation were also identified as independent prognostic factors (all *p* < 0.05)(Table 3).

**Table 3:** Multivariable binomial logistic regression results for predicting occult LN metastasis

Parameters	Coefficient (β)	S.E.	OR	95% CI for OR	*p*-value
ECV grade	1.138	0.539	3.119	(1.084, 8.978)	0.035
Tumor area	0.054	0.017	1.055	(1.020, 1.092)	0.002
WHO types	0.771	0.380	2.161	(1.025, 4.555)	0.043
Differentiation	−0.783	0.305	0.457	(0.251, 0.831)	0.010
Hematocrit	−0.011	0.036	0.989	(0.922, 1.062)	0.766

*LN* lymph node, *S.E.* standard error, *OR* odds ratio, *CI* confidence interval, *ECV* extracellular volume

**Fig. 5:**
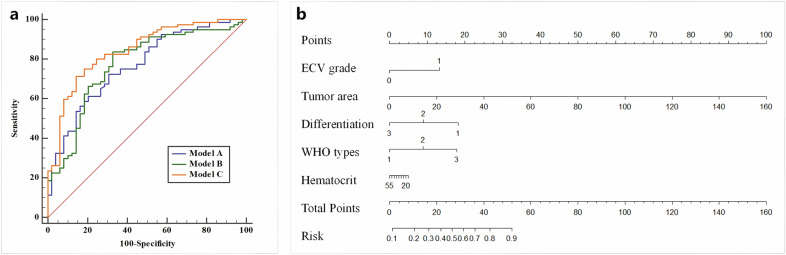
**a** Receiver operating characteristic curve analysis of different models to predict occult LN metastasis. The areas under the curves of the Model A, Model B, and Model C were 0.768, 0.774, and 0.843, respectively. **b** A nomogram based on the multivariate logistic regression model to predict occult LN metastasis, by integrating the features of ECV grade, tumor area, differentiation, WHO types, and hematocrit. Model A, the clinicopathological model, by integrating WHO types, differentiation degree, and hematocrit. Model B, the CT parameters model, by integrating ECV grade and tumor area; Model C, the combined model, by integrating clinicopathological and CT parameters. LN, lymph node; ECV, extracellular volume

As presented in Table 4, the clinicopathological model (Model A) yielded an AUC of 0.768 by combining WHO types, differentiation degree, and hematocrit. The CT parameters model (Model B) integrated ECV grade and tumor area, and the AUC value was 0.774. The combined model (Model C), integrating the clinicopathological and CT parameters, yielded an AUC of 0.843 (Fig. 5a). In the DeLong test (Table 5), significant differences were revealed in the AUCs of Model C vs. Model A (*p* = 0.013), and Model C vs. Model B (*p* = 0.020). No statistical significance was seen between the AUCs of Model A vs. Model B (*p* = 0.905).

**Table 4:** The diagnostic performance of different models to predict occult LN metastasis

Models	AUC (95% CI)	Accuracy (95% CI)	Sensitivity (95% CI)	Specificity (95% CI)	*p*-value
Model A	0.768 (0.686–0.838)	0.713 (0.626–0.789)	0.725 (0.606–0.824)	0.694 (0.545–0.817)	< 0.001*
Model B	0.774 (0.693–0.843)	0.776 (0.691–0.847)	0.838 (0.738–0.910)	0.674 (0.525–0.800)	< 0.001*
Model C	0.843 (0.769–0.901)	0.768 (0.710–0.859)	0.713 (0.600–0.808)	0.857 (0.728–0.941)	< 0.001*

Model A: the clinicopathological model, by integrating WHO types, differentiation degree, and hematocrit. Model B: the CT parameters model, by integrating ECV grade and tumor area. Model C: the combined model, by integrating clinicopathological and CT parameters

*AUC* area under the curve, *CI* confidence interval, *LN* lymph node, *ECV* extracellular volume

* *p* < 0.05 with receiver operating characteristic curve analysis

**Table 5:** The DeLong test of AUCs comparison for different diagnostic models

Models comparison	AUC difference	*Z* value	*p*-value
Model A vs. Model B	0.007	0.120	0.905
Model C vs. Model A	0.075	2.475	0.013*
Model C vs. Model B	0.069	2.335	0.020*

Model A: the clinicopathological model, by integrating WHO types, differentiation degree, and hematocrit. Model B: the CT parameters model, by integrating ECV grade and tumor area. Model C: the combined model, by integrating clinicopathological and CT parameters

*AUC* area under the curve, *ECV* extracellular volume

* *p* < 0.05 with the DeLong test

A nomogram was constructed based on the combined model to predict occult LN metastasis (Fig. 5b). To further validate the clinical utility of the combined model, the decision-curve analysis and calibration plot were performed in Fig. S1.

### Recurrence risk stratification

Of the 129 patients, 102 patients had complete postoperative follow-up data, and the overall recurrence rate was 20.6% (21/102) with a median RFS of 17 months. Among the 21 recurrent cases, 15 patients had peritoneal recurrence, while 6 patients had non-peritoneal recurrence. The median RFS of the N (+) group was significantly shorter than that of the N (−) group (14 months vs. 20 months, *p* < 0.001).

**Fig. 6:**
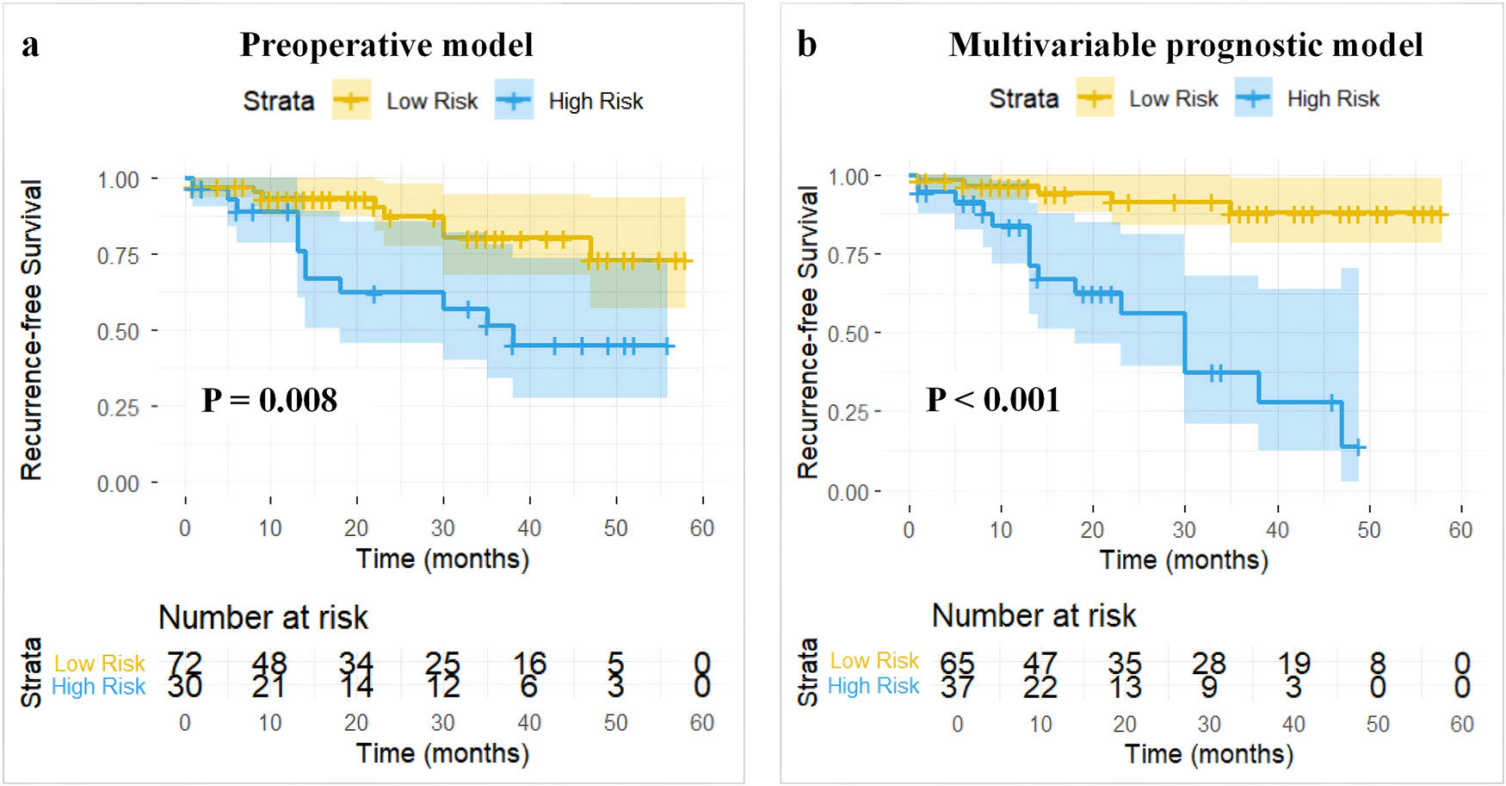
The Kaplan–Meier curves of recurrence-free survival scaled by the preoperative model (**a**) and multivariable prognostic model (**b**) with high-risk and low-risk groups

Based on the optimal risk cutoff score of 0.82, the preoperative combined model was stratified into high-risk and low-risk groups, with significantly different median RFS (14 months vs. 16 months, *p* = 0.008). The Kaplan–Meier curve of the preoperative combined model is displayed in Fig. 6a. Bootstrap validation (1000 repetitions) showed a minimal decrease in the C-index (0.004), indicating low overfitting and good model generalizability within the cohort (original C-index: 0.638; optimism-corrected C-index: 0.634; optimism: 0.004).

Multivariable Cox regression analysis was performed, incorporating both the preoperative model and postoperative pathological features. Postoperative pathological features with significant differences between the N (−) and N (+) groups were included in the multivariate Cox regression, including T stage, LVI, PNI, Lauren classification, and differentiation. The results demonstrated that the preoperative model remained a significant independent predictor of RFS (HR = 3.095, 95% CI: 1.099–8.719, *p* = 0.032). Pathological T stage was also identified as an independent prognostic factor (HR = 5.576, 95% CI: 1.237–25.135, *p* = 0.025) (Table S1).

Subsequently, we developed a multivariable prognostic model for RFS based on the Cox regression results. Bootstrap validation (1000 repetitions) showed acceptable overfitting and good discriminative ability (original C-index, 0.756; optimism-corrected C-index, 0.724; optimism, 0.032). Based on the optimal risk cutoff score, the Kaplan–Meier curve for the multivariable prognostic model is presented in Fig. 6b (*p* < 0.001).

### Interobserver agreement

The ECV fraction and tumor area demonstrated ICCs of 0.870 (95% CI: 0.821–0.907) and 0.891 (95% CI: 0.741–0.943), respectively, indicating good consistency within the two radiologists. We further conducted Bland–Altman analysis to estimate measurement bias and limits of agreement, displaying good interobserver agreement (Fig. 3c, d).

## Discussion

In this study, the proposed CT-based ECV model demonstrated favorable diagnostic performance for preoperative detection of occult LN metastasis (AUC = 0.843) and revealed a significant difference in stratifying postoperative recurrence risk in GC (*p* = 0.008).

Our findings revealed that the N (+) group exhibited significantly higher proportions of poorly cohesive carcinoma and poor differentiation based on endoscope pathology. Additionally, the N (+) group demonstrated significantly higher CT-measured tumor area and ECV fraction, and significantly lower hematocrit levels. The combined model achieved an AUC of 0.843 for predicting occult LN metastasis, significantly outperforming models based on clinical-pathological or CT parameters alone. The preoperative combined model with the high-risk group revealed a significantly shorter median RFS compared to the low-risk group.

Compared with gastric tubular adenocarcinoma, poorly cohesive carcinomas are characterized by loose intercellular adhesion and a higher risk of infiltration [27, 28], which are prone to disseminate from the primary tumor and facilitate lymphatic dissemination [12, 14]. Kao et al [14] demonstrated that signet ring cell carcinoma had more LN metastasis compared to non-signet ring cell carcinoma in T2-T4 GCs (*p* < 0.001). Our results demonstrated a significantly higher proportion of poorly cohesive carcinoma in the N (+) group, which was consistent with previous studies. Besides, poorly differentiated GCs are more aggressive and frequently exhibit heightened metastatic potential. A previous study showed a significantly higher LN metastasis in poorly differentiated GCs [29], consistent with our results. Previous studies have revealed that stromal TILs are associated with the clinical prognosis of tumor patients [19, 30]. Notably, no significant difference in stromal TILs percentage between the two groups was seen in our study, possibly suggesting the limited value of stromal TILs for predicting occult LN metastasis. Furthermore, a significantly lower hematocrit level was observed in the N (+) group. GC is a cachectic disorder frequently complicated by chronic blood loss or prolonged malnutrition, which may progressively lead to anemia and decreased hematocrit levels. GC patients with LN metastasis may have a longer disease duration and may be more prone to reduced hematocrit levels.

ECV fraction has emerged as a quantitative biomarker for evaluating ECM volume. ECM is an important part of the tumor microenvironment, which is the main driving force of malignant tumor formation and plays a pivotal role in tumor invasion and metastasis [7, 31]. A significantly higher ECV fraction in the N (+) group may be mainly attributed to the following two points. Firstly, GC with LN metastasis tends to be more aggressive and exhibits abundant intratumoral neovascularization. However, these neovessels are characterized by structural immaturity and fragile vascular wall [32], contributing to elevated microvascular perfusion and enhanced vascular permeability [10, 33]. Consequently, it increased contrast agent entry into the tissue space and elevated ECV fraction. In addition, GC with LN metastasis may recruit more inflammatory cell infiltration and persistent activation of cancer-associated fibroblast in the tumor microenvironment, promoting ECM deposition and resulting in increased ECV fraction [10].

Indeed, previous studies have applied CT-based ECV to evaluate various neoplasms [6, 7, 9–11, 33–35]. Most of the previous studies [7, 10, 11, 33, 34] showed that the more aggressive tumors had higher ECV fraction, consistent with our results. Zhang et al [10] reported that CT-based ECV fraction was significantly higher in LVI and PNI positive group of colorectal cancer (*p* = 0.001). Hu et al [11] revealed that the ECV fraction was significantly lower in the less aggressive microsatellite instability (MSI) group of GC (*p* = 0.002 and 0.008 in the training and validation groups, respectively). Liu et al [34] found the ECV-higher group was a significant risk factor associated with shorter recurrence-free survival and shorter overall survival in bladder cancer (*p* < 0.001). Additionally, we revealed that the N (+) group had a significantly higher CT-measured tumor area. The possible reason was that the larger tumors tend to have a longer disease duration, resulting in a greater risk of LN metastasis.

By integrating CT parameters (including ECV) and clinicopathological features, we established the clinicopathological model, CT parameters model, and combined model, with AUCs of 0.768, 0.774, and 0.843, respectively. The combined model demonstrated a significantly enhanced diagnostic efficiency compared to the CT model and clinicopathological model (both *p* < 0.05). We further constructed a nomogram, decision-curve analysis, and calibration plot of the combined model, which facilitated the clinical application. In addition, we also performed a prognostic analysis on the combined model to further validate the clinical value, suggesting the high-risk group had a significantly short median RFS. Furthermore, the preoperative prognostic model remained an independent predictor of RFS after adjusting for postoperative pathological confounders. These findings suggested that the combined model may be beneficial to early prediction of occult LN metastasis and early identification of recurrence in GC patients, which can provide some reference values for clinical diagnosis and treatment.

This study had several limitations. First, this retrospective study had a relatively small sample size with a single center, and larger sample and multicenter cohorts are needed for further validation. Second, the calculation of ECV values used DP images in our study, but there was no uniform standard for the optimal enhanced time for calculating ECV. Most studies showed that the equilibrium phase of 3 min was a good scanning period for evaluating ECV [11, 36], which was adopted in our study. Finally, prognostic assessment only used RFS and did not include overall survival; we would explore it in future studies.

In conclusion, the proposed preoperative combined model, integrating CT parameters (including ECV) and endoscopic pathological features, exhibited a promising performance for early predicting occult LN metastasis and stratifying postoperative recurrence risk. Those findings may provide valuable references for clinical diagnosis and treatment in GCs.

## Supplementary information

Supplementary information

## Data Availability

The datasets used and analyzed during the current study are available from the corresponding author on reasonable request.

## Abbreviations

AUC: Area under the curve
DP: Delayed phase
ECV: Extracellular volume
GC: Gastric cancer
ICC: Intraclass correlation coefficient
LN: Lymph node
NLR: Neutrophil-lymphocyte ratio
PLR: Platelet-lymphocyte ratio
RFS: Recurrence-free survival
ROI: Region of interest
SII: Systemic immune-inflammation index
TILs: Tumor-infiltrating lymphocytes
