# Supplementary material for: Predictive value of multivariate models combining CT-based extracellular volume fraction with clinicopathological parameters for preoperative detection of occult lymph node metastasis in gastric cancer
Source: Insights Imaging. 2025 Dec 22;16:285. doi: 10.1186/s13244-025-02172-6 (PMC12722588; doi:10.1186/s13244-025-02172-6)
Supplement: Supplementary file 1 — Supplementary information [file 13244_2025_2172_MOESM1_ESM.pdf]

Predictive value of multivariate models combining CT-based extracellular volume fraction with clinicopathological parameters for preoperative detection of occult lymph node metastasis in gastric cancer

ELECTRONIC SUPPLEMENTARY MATERIAL

Hematological indices

Hematological indices, including hematocrit, absolute lymphocyte, neutrophil, and platelet counts were collected. Then, the neutrophil-lymphocyte ratio (NLR) was calculated with the absolute neutrophil and lymphocyte counts. Platelet-lymphocyte ratio (PLR) was gained by the absolute platelet and lymphocyte counts. Systemic immune-inflammation index (SII) was obtained by platelet count × NLR.

CT image acquisition

The CT examination covered the upper or entire abdomen. Following the unenhanced scan, iodinated contrast agent (Omnipaque 350 mg I/mL, GE Healthcare) was injected using a high-pressure syringe at a rate of 3.0 mL/s and a dose of 1.5 mL/kg. The arterial phase, venous phase, and delayed phase were obtained after 40 s, 70 s, and 180 s after contrast agent injection, respectively. The CT scan parameters were: tube voltage 100-120 kV, tube current 150-250 mA, field of view 35-50 cm, matrix 512 × 512, rotation time 0.7 s, and pitch 1.0875.

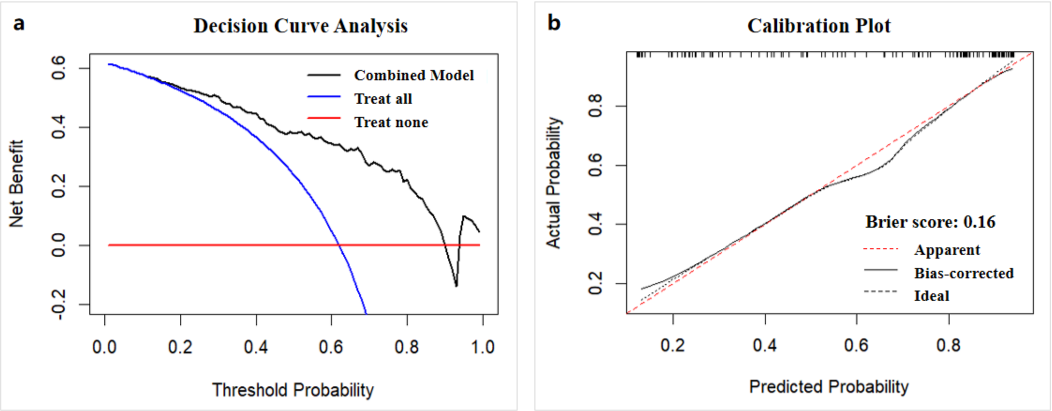

Fig. S1 Decision curve analysis (**a**) and calibration plot (**b**) of the combined model for predicting occult lymph node (LN) metastasis. **(a)** The y-axis represents the net benefit, and x-axis represents threshold probability. The combined model (black solid line) demonstrates a higher net benefit across the majority of the range of threshold probabilities, compared to treating all patients (blue dashed line) or treating none (red dotted line). **(b)** The y-axis indicates the actual probability of LN metastasis, and x-axis indicates the predicted probability. The dashed diagonal line represents perfect calibration. The combined model (solid line) shows good calibration with the ideal line, with a Brier score of 0.16.

Table S1. Multivariate Cox analysis for recurrence-free survival of GC patients

Variable	HR	CI_lower	CI_upper	*P* value
Preoperative model	3.095	1.099	8.719	**0.032***
T stage	5.576	1.237	25.135	**0.025***
LVI	1.360	0.489	3.781	0.556
PNI	1.065	0.260	4.356	0.930
Lauren classification (1)	0.859	0.189	3.918	0.845
Lauren classification (2)	3.792	0.949	15.155	0.059
Differentiation	3.339	0.605	18.419	0.166

GC, gastric cancer; *HR*, hazard ratio; *CI*, confidence interval; *LVI*, lymphovascular invasion; *PNI*, perineural invasion.
